# AI-Enabled Precision Echocardiography: Toward Personalized Cardiovascular Care

**DOI:** 10.3390/diagnostics16050694

**Published:** 2026-02-26

**Authors:** Lamia Al Saikhan

**Affiliations:** Department of Cardiac Technology, College of Applied Medical Sciences, Imam Abdulrahman Bin Faisal University, Dammam 34212, Saudi Arabia; lkalsaikhan@iau.edu.sa

**Keywords:** precision medicine, artificial intelligence, precision echocardiography, cardiovascular phenotyping, personalized cardiovascular care

## Abstract

Conventional echocardiography traditionally relies on population-derived reference values and dichotomous classification schemes that do not fully account for individual patient characteristics or disease complexity. Artificial intelligence (AI) is driving a paradigm shift toward precision echocardiography by enabling patient-specific cardiovascular assessment through the integration of phenotypic, clinical, and biological data. This review examines how AI is transforming echocardiography from a population-based test into a patient-specific assessment tool that supports precision cardiovascular care. It highlights three clinical applications with potential clinical impact: Heart Failure with Preserved Ejection Fraction phenogrouping for targeted therapy selection, cardio-oncology surveillance with individualized cardiotoxicity risk prediction, and cardiomyopathy risk stratification for personalized sudden cardiac death prevention. For each application, it describes the clinical challenge, the AI-enabled precision solution, and its potential clinical impact. It also outlines a practical roadmap for clinical adoption. Precision echocardiography, powered by AI, holds promise for transforming cardiovascular imaging and diagnostics by enabling more patient-specific assessment, earlier disease detection, and personalized therapeutic strategies.

## 1. Introduction

Conventional echocardiography traditionally relies on population-derived reference values and dichotomous classification schemes that fail to fully account for individual patient characteristics and disease complexity. For example, a “normal” left ventricular ejection fraction (LVEF) ranges from 52% to 72% for males (or 54% to 74% for females), a 20-percentage-point span that reflects substantial physiological heterogeneity [1]. Yet identical diagnostic thresholds are often applied to a 22-year-old competitive athlete and a 72-year-old patient with hypertension and diabetes, despite their fundamentally different cardiovascular phenotypes. This one-size-fits-all approach can overlook subclinical disease, yield imprecise risk stratification, and constrain personalized treatment selection [2,3]. The clinical consequences are substantial and include delayed diagnosis after irreversible myocardial damage, suboptimal therapeutic decision-making, and missed opportunities for early intervention in high-risk individuals.

Precision medicine has revolutionized cancer care by enabling targeted therapies guided by tumor genetics, improving outcomes in selected subgroups [4,5]. In contrast, cardiovascular medicine has lagged behind, relying largely on population-based risk models, scores and treatment algorithms. Echocardiography generates a wealth of phenotypic data, yet conventional analysis typically extracts only approximately 10 to 20 basic measurements from images that contain orders of magnitude more information [6,7]. Recent advancements in artificial intelligence (AI) offer an unprecedented opportunity to address this limitation through comprehensive extraction of imaging phenotypes and their integration into a truly patient-specific cardiovascular assessment [8,9].

Four capabilities position AI as uniquely suited to enabling precision echocardiography. First, AI enables comprehensive phenotyping by extracting hundreds of quantitative features, including myocardial texture patterns, regional deformation characteristics, and geometric remodeling indices that extend beyond human perceptual capacity [10,11]. Second, AI can improve reproducibility by reducing the variability inherent to conventional echocardiographic measurements, which is essential for precise longitudinal tracking of individual patients [1,12,13,14]. Third, AI excels at pattern recognition, enabling identification of subtle individual-specific signatures and disease-related phenotypes that distinguish a patient’s physiological “normal” from early pathological change [15,16,17,18]. Fourth, AI facilitates multimodal integration by combining echocardiographic phenotypes with electrocardiographic data, circulating biomarkers, clinical characteristics, and genetic information to generate comprehensive cardiovascular profiles that support personalized risk assessment and treatment selection [19,20,21,22].

This review examines how AI is transforming echocardiography from a population-based test into a patient-specific assessment tool that supports precision cardiovascular care. Three clinical applications with potential clinical impact are highlighted: HFpEF phenogrouping for targeted therapy selection, cardio-oncology surveillance with individualized cardiotoxicity risk prediction, and cardiomyopathy risk stratification for personalized sudden cardiac death prevention. For each application, it describes the clinical challenge, the AI-enabled precision solution, and its potential clinical impact. It also outlines a practical roadmap for clinical adoption. Precision echocardiography, powered by AI, holds promise for transforming cardiovascular imaging and diagnostics by enabling patient-specific assessment, earlier disease detection, and personalized therapeutic strategies.

In preparing this review, a comprehensive literature search was performed across PubMed/MEDLINE, Web of Science, and Google Scholar databases to identify advances in AI-enabled precision echocardiography. The search strategy incorporated the following keywords: “artificial intelligence” OR “machine learning” OR “deep learning” AND “echocardiography” OR “echocardiogram” AND “heart failure” OR “HFpEF” OR “cardiotoxicity” OR “cardiomyopathy” OR “precision medicine” OR “personalized medicine” OR “phenotyping”. In addition, a snowball searching approach was employed to further identify relevant publications through reference lists of key articles and reviews. Of the more than 120 articles initially retrieved, 80 were included in this review based on their relevance and scientific methodological quality.

## 2. From Population-Derived Averages to Patient-Specific Phenotypes

### 2.1. Precision Echocardiography Paradigm

Traditional echocardiography evaluates patients against population-derived reference values and fixed diagnostic cutoffs, a paradigm that does not capture substantial inter-individual variability, assigning identical “normal” interpretations to clinically distinct patients [1,2,3]. Precision echocardiography reverses this paradigm by emphasizing intra-individual comparisons rather than evaluation against population averages [8,9]. The conceptual framework for precision echocardiography was articulated by Shah, who described echocardiography as a “digital biopsy” capable of deconstructing cardiovascular biology at the level of the individual patient [23]. Accordingly, the central question becomes “How does the patient compare to their own baseline?” rather than “How does the patient compare to the population average?”.

**Figure 1:**
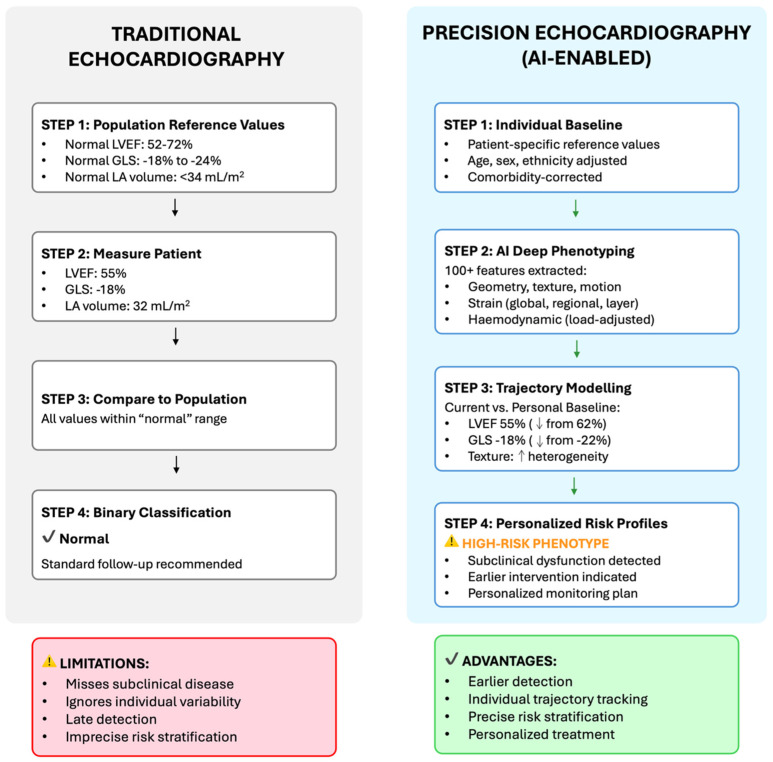
From Population-Derived to Patient-Specific Cardiovascular Assessment. This figure compares and contrasts traditional echocardiography with precision echocardiography (AI-enabled). Traditional echocardiography looks at individual patients in relation to population-derived reference values, resulting in binary normal/abnormal classification that can miss subclinical disease and individual variability. Precision echocardiography uses AI to establish patient-specific baselines, extract comprehensive phenotypic features beyond conventional metrics, model individual trajectories over time, and generate personalized risk profiles that enable earlier detection and targeted interventions.

This approach establishes patient-specific reference values, uses AI to extract comprehensive phenotypic features, monitors longitudinal trajectories within individuals, and integrates multimodal clinical data to construct personalized risk profiles (Figure 1) [10,11,12,15,16,17,18,19,20].

### 2.2. AI-Enabled Deep Phenotyping

Using AI, hundreds of quantitative features can be extracted from echocardiographic images—far beyond the approximately 15–20 conventional measurements typically analyzed and reported by human operators. To illustrate this capability, consider the following representative clinical example.

*Traditional Echocardiography Report:*

LVEF: 55%.

Left atrial volume: 34 mL/m^2^.

E/e′ ratio: 12.

*Conclusion: Normal LV systolic and diastolic function.*
*AI-Enabled Precision Echocardiography Report (Same Patient):*

LVEF: 55% (within population-defined normal range; a 7-percentage-point decline from the patient’s personal baseline of 62% measured three years ago).

Left atrial volume: 34 mL/m^2^ (at the upper limit of normal reference values).

E/e′ ratio: 12 (elevated relative to hemodynamic status/loading conditions).

Global longitudinal strain (GLS): −16% (reduced compared with the age-expected value of −20%; predictive of adverse cardiovascular events) [24].

Myocardial texture analysis: increased heterogeneity, consistent with early interstitial fibrosis.

Regional strain pattern: relative apical sparing (strain ratio 0.71), a pattern associated with infiltrative cardiomyopathy [25].

*Interpretation: Subclinical LV dysfunction with high-risk phenotypic features. Further evaluation is recommended, including cardiac MRI and biomarker testing to assess for infiltrative disease.*

This contrast, which only exploits a subset of available echocardiography information, highlights the distinction between population-based and precision approaches to echocardiography [26,27]. While the traditional report may fail to identify early disease due to reliance on binary classification against broad population reference ranges, the precision report detects subclinical disease through individualized trajectory analysis, deep phenotyping, and disease-specific pattern recognition.

### 2.3. Key Components of Precision Phenotyping

AI-enabled phenotyping relies on four synergistic components.

Baseline individualization defines patient-specific reference values that account for age, sex, ethnicity, athletic conditioning, body size and composition, comorbidities, and other predisposing factors. This personalization is critical for distinguishing adaptive features such as appropriate remodeling, e.g., physiological hypertrophy in elite athletes, from maladaptive or pathological hypertrophy in sedentary individuals, distinctions that population-based cutoffs may not capture.

Comprehensive feature extraction integrates geometric indices, such as sphericity and remodeling patterns; myocardial functional parameters, including global, regional, and layer-specific strains; tissue characteristics, such as texture heterogeneity and reflectivity patterns; hemodynamic parameters, including load-adjusted function; and temporal features, including motion trajectory and contraction synchrony. Together, these dimensions can generate a patient-specific cardiovascular fingerprint [10,11,15,16,17,18].

Longitudinal trajectory modelling compares current measurements with patient-specific baselines and predicted trajectories, allowing detection of clinically meaningful changes even when values remain within population-defined “normal” ranges. For example, a decline in GLS from −22.5% to −18.5% represents an 18% relative reduction in myocardial function that may precede overt heart failure, despite both values falling within the conventional normal range of −18% to −24% [24].

Multimodal integration combines echocardiographic phenotypes with electrocardiographic patterns, biomarkers, genetic information, and clinical characteristics. This integrative approach has outperformed single-modality approaches across multiple cardiovascular applications, including heart failure risk prediction, cardiotoxicity forecasting, and sudden cardiac death risk stratification [9,20,26].

### 2.4. Clinical Implications of Precision Phenotyping

**Figure 2:**
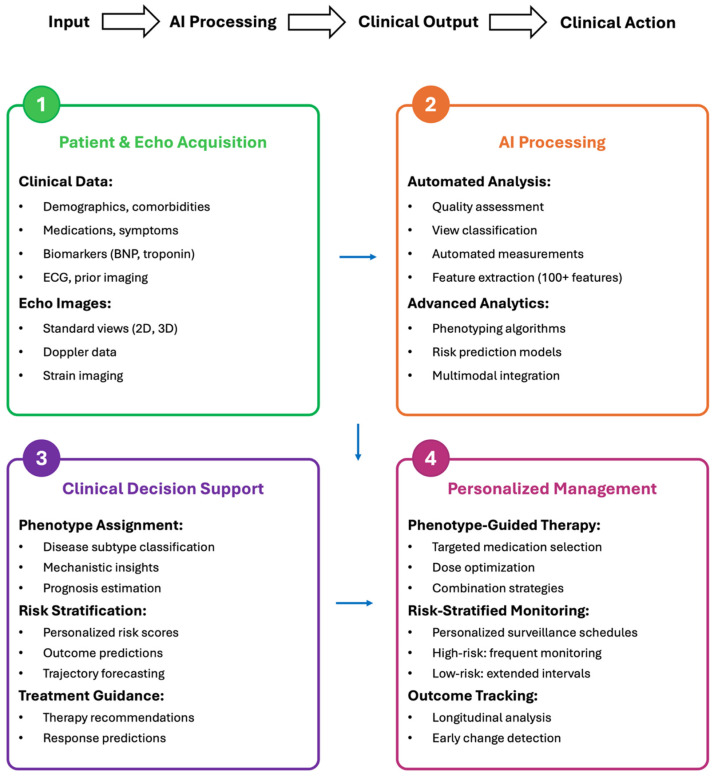
AI Workflow in Clinical Practice Echocardiography. An AI-enabled precision echocardiography workflow can integrate into clinical practice through four sequential steps. In Step 1 (Input), patient clinical data and echocardiographic images are acquired using standard protocols. In Step 2 (AI Processing), automated quality assessment, view classification, and measurement extraction are followed by advanced analytics including phenotyping algorithms, risk prediction models, and multimodal data integration, extracting more than 100 quantitative features beyond human perceptual capacity. In Step 3 (Clinical Decision Support), AI generates phenotype assignments that identify disease subtypes and mechanisms, personalized risk scores that predict outcomes, and treatment guidance recommending therapies with the highest likelihood of benefit. In Step 4 (Personalized Management), clinicians implement phenotype-guided therapy selection, risk-stratified monitoring schedules, and longitudinal trajectory tracking for early change detection. This workflow can transform echocardiography from descriptive imaging into a precision diagnostic and therapeutic decision-support tool.

Precision phenotyping offers several clinical benefits. It enables earlier detection of subclinical disease, before conventional criteria identify abnormalities; supports personalized treatment selection; improves risk stratification through AI-derived phenotypes that enhance discrimination for adverse cardiovascular outcomes [27]; and optimizes individual monitoring through risk-based surveillance that focuses resources where they are most likely to be beneficial. Ultimately, echocardiography may be transformed into a precision tool that characterizes individual phenotypes, predicts patient trajectories, and guides individualized treatment. Figure 2 illustrates an AI-enabled precision echocardiography workflow integrated into clinical practice, encompassing sequential steps from data acquisition to personalized patient management.

## 3. Clinical Applications

### 3.1. HFpEF

#### 3.1.1. The HFpEF Challenge

Heart failure with preserved ejection fraction (HFpEF) is a prevalent and therapeutically challenging phenotype, comprising more than half of all heart failure patients [28,29]. In contrast to heart failure with reduced ejection fraction (HFrEF), HFpEF has shown limited responsiveness to conventional therapies, underscoring the need for precision-guided, individualized treatment strategies [28,29,30]. Mortality benefits of most medications effective in HFrEF have not been demonstrated in HFpEF in large randomized clinical trials [31,32], although SGLT2 inhibitors have recently shown consistent, though modest, benefits [33,34]. HFpEF heterogeneity is considered the primary reason for therapeutic ineffectiveness [35,36]. This complex systemic syndrome encompasses a spectrum of pathophysiological mechanisms, including atrial myopathy, pressure-overload-mediated diastolic dysfunction, and infiltrative myocardial processes [37]. Clinical trials that enroll heterogeneous HFpEF populations can dilute treatment effects, such that therapies conferring substantial benefit in specific subgroups may yield only modest average effects at the overall sample level [38].

#### 3.1.2. Precision Solution (AI-Enabled Phenogrouping)

AI-enabled phenogrouping addresses HFpEF heterogeneity by identifying pathophysiologically distinct subgroups [18,39,40,41,42]. In a retrospective analysis of the prospective KaRen cohort, Hedman et al. applied unsupervised machine learning to 32 echocardiographic variables and 11 laboratory and clinical variables, identifying six distinct phenogroups in 320 HFpEF patients [41]. These phenogroups demonstrated pronounced heterogeneity in clinical profiles, comorbidities, disease severity, and outcomes. Composite event rates of heart failure hospitalization and all-cause mortality ranged from 17% to 61% at 1.5 years of follow-up, reflecting more than a threefold difference between the lowest-risk subgroup (phenogroup 3) and the highest-risk subgroup (phenogroup 2) [41]. While this retrospective observational evidence is hypothesis-generating, the study highlights the potential of AI-powered echocardiographic phenotyping to refine risk stratification and support individualized clinical management in HFpEF. Kobayashi et al. applied machine learning to echocardiographic data from the HOMAGE trail, identifying distinct cardiac structural and functional phenotypes with differential responses to spironolactone therapy [42]. A recent systematic review by Epelde synthesize the expanding but methodologically heterogenous evidence base for AI-enabled HFpEF phenotyping [40]. Yet prospective validation studies and randomized trails evaluating clinical utility remain limited, representing critical next steps for clinical translation of AI-enabled HFpEF phenogrouping.

#### 3.1.3. Clinical Impact

The implications of AI-enabled HFpEF phenogrouping extend beyond risk stratification to support more precise therapeutic decision-making [39,40]. Phenotype-guided therapy can enable clinicians to tailor treatment according to the underlying pathophysiology rather than applying guideline-recommended therapies uniformly, a strategy that may dilute subgroup-specific responses [38,41,42]. This precision-prescribing approach has the potential to increase treatment efficacy and improve clinical trial efficiency by selectively enrolling patients most likely to benefit from targeted interventions [39,40,41,42]. However, integration of HFpEF phenogrouping into routine echocardiography workflows will require validation across diverse populations, standardization of phenogrouping algorithms, and prospective evidence demonstrating improved patient outcomes. Prospective trails are also needed to determine whether AI-enabled, phenogrouping-guided therapy can meaningfully improve clinical outcomes before routine implementation.

### 3.2. Cardio-Oncology

#### 3.2.1. The Cardiotoxicity Challenge

As cancer survival improves, cancer therapy-related cardiac dysfunction (CTRCD) has become a growing clinical challenge [43,44]. Anthracyclines induce dose-dependent cardiotoxicity influenced by cumulative exposure and patient-specific risk factors [44,45,46]. Other cancer therapies also carry cardiovascular risks. Historically, cardiotoxicity monitoring has relied on serial echocardiographic assessment of LVEF, with intervention typically triggered by a decline of at least 10 percentage points resulting in LVEF below normal thresholds. This approach has three key limitations. It can detect cardiotoxicity late, when myocardial injury may already be irreversible [47]. It applies uniform surveillance despite substantial inter-individual variation in risk [48,49]. It can also be inefficient, requiring frequent echocardiography for all patients regardless of individual risk profile [50].

#### 3.2.2. Precision Solution (Risk-Stratified, Trajectory-Based Monitoring)

AI-enabled cardio-oncology addresses these limitations through a three-step framework [20,51]:

Step 1: Baseline AI-Based Risk Stratification.

Prior to cancer therapy, AI models integrate baseline echocardiographic phenotypes (e.g., LVEF, GLS, and diastolic function parameters), clinical characteristics (e.g., age and cardiovascular risk factors), planned treatment (e.g., drug type and cumulative dose), and/or genetic markers to predict individual cardiotoxicity risk [20,51,52,53]. In a prospective cohort study of 211 breast cancer patients receiving anthracycline therapy, Chang et al. developed machine learning models to predict CTRCD and HFrEF over a 3-year follow-up period, achieving AUC of 0.66 and 0.81, respectively [53]. While this study demonstrates the feasibility of AI-based risk stratification, external validation and clinical implementation studies remain limited.

Step 2: Personalized Monitoring Intensity.

AI-enabled risk stratification allows individualized surveillance as follows: high-risk patients undergo echocardiography at each chemotherapy cycle, receive prophylactic cardioprotective therapy, and have AI-generated individual trajectory models; medium-risk patients follow standard monitoring; and low-risk patients undergo reduced-intensity monitoring. This personalized approach has the potential to reduce imaging burden and enhance early cardiotoxicity detection, but rigorous clinical implementation studies demonstrating these benefits are needed.

Step 3: Individual Trajectory Tracking.

AI models predict expected changes in LVEF and/or GLS based on baseline values, treatment exposure, and patient-specific risk factors, generating a personalized expected trajectory. Serial echocardiograms are evaluated against this predicted trajectory rather than population-based norms. Deviations beyond expected ranges can potentially trigger early intervention even when absolute values remain within population-defined normal ranges [21].

Ahmadi et al. reported that radiomics analysis of baseline 2D echocardiography predicted 12-month post-chemotherapy LVEF decline with 92% accuracy in a retrospective cohort, identifying high-risk patients before conventional criteria would detect cardiotoxicity [21]. This study demonstrates technical feasibility of trajectory prediction. For example, a patient with a baseline GLS of −22.5% declining to −19.5% at cycle 3 (both “normal”) would not meet traditional intervention thresholds. AI trajectory modelling, however, would interpret this 13% relative decline as clinically meaningful and could trigger earlier intervention. However, before routine clinical implementation, robust prospective studies are required to determine whether AI-guided early interventions lead to improved patient outcomes compared to standard monitoring protocols.

#### 3.2.3. Clinical Impact

Emerging evidence from studies of AI-enabled cardio-oncology suggests potential clinical benefits across multiple dimensions [20,21,51,52,54]. Earlier detection of cardiotoxicity, before conventional criteria are met, can enable timely intervention, while myocardial injury remains potentially reversible [21]. Targeted surveillance of high-risk patients can reduce unnecessary monitoring in low-risk individuals and may reduce overall echocardiography volume. Fewer premature cancer therapy discontinuations may support improved treatment completion and better oncologic outcomes. Personalized risk communication may also enhance patient satisfaction and support informed shared decision-making regarding cancer treatment and surveillance strategies. However, rigorous prospective validation and implementation studies are required before these methods can be routinely adopted in clinical practice, as whether AI-enabled early cardioprotective intervention prevents heart failure or improves cardiovascular outcomes remains to be explored.

### 3.3. Cardiomyopathy

#### 3.3.1. The Sudden Cardiac Death Prediction Challenge

Hypertrophic cardiomyopathy (HCM), a common inherited cardiac disorder, is associated with sudden cardiac death (SCD) due to ventricular arrhythmias [55,56]. Implantable cardioverter–defibrillators (ICDs) are effective in preventing SCD but carry risks and complications, including lead-related complications, infection, and inappropriate shocks [57]. Precise risk stratification is crucial for identifying patients likely to benefit from ICD therapy while avoiding unnecessary device implantation. Current risk stratification relies on structured clinical risk scores that integrate clinical and echocardiographic variables [58,59]. However, these scores demonstrate only modest discrimination, resulting in missed high-risk patients or unnecessary ICD implantation in low-risk individuals [60,61]. A fundamental limitation is that conventional risk models capture only a fraction of the phenotypic and genotypic complexity underlying arrhythmic risk [59].

#### 3.3.2. Precision Solution (AI-Enabled Risk Prediction)

AI-enabled risk stratification integrates comprehensive echocardiographic phenotypes with clinical, biomarker, and genetic data [19,22,62,63,64]. Emerging evidence suggests that machine-learning models derived from multimodal approaches may offer improved discriminatory performance compared with traditional risk scores for ventricular arrhythmias and SCD [22,58,59,63]. In a proof-of-concept study, Enayati et al. applied unsupervised deep learning clustering to comprehensive echocardiographic datasets and identified distinct phenotypic subgroups with varying arrhythmic risk profiles that could inform ICD implementation decisions, demonstrating the utility of data-driven phenotype discovery [63]. More recently, in a retrospective analysis of JHH-HCM and SHVI-HCM registries, Lai et al. developed a multimodal deep-learning model integrating echocardiography, MRI, and clinical data that demonstrated improved identification of HCM patients at risk of SCD [22]. This performance advantage is attributed to the ability of AI-enabled phenotyping to capture complex multidimensional features that may predict arrhythmic risk beyond conventional echocardiographic and clinical parameters. Analyses of strain patterns, myocardial texture, and geometric phenotypes may collectively identify higher-risk characteristics, such as mechanical dispersion, septal-dominant hypertrophy, and diffuse fibrosis, enabling more precise risk stratification for ventricular arrhythmias and SCD [22,65]. Future prospective validation studies and randomized trials comparing AI-enabled risk stratification versus guideline-based approaches for ICD implementation decision-making are needed to establish clinical utility.

#### 3.3.3. Clinical Impact

Improved discrimination from AI-enabled risk prediction has the potential to transform SCD prevention in HCM across multiple clinical and patient-centered dimensions. Better risk stratification can identify high-risk patients who may be overlooked by conventional scoring systems, supporting more personalized decision-making [58,60,61,63]. AI-based models may also help avoid unnecessary ICD implantation in low-risk patients while clarifying risk in borderline cases, potentially reducing device-related complications while preventing SCD [57,63]. Moreover, phenotype-informed decision-making may support shared decision-making grounded in patient-specific data rather than population-average risk estimates.

## 4. Implementation: From Research to Practice

### 4.1. Current Clinical Availability

Translation of precision echocardiography from research into clinical practice is progressing rapidly, with several commercial AI platforms holding FDA clearance or CE marking [66,67,68]. Caption Health provides AI-guided imaging that can improve image quality and reduce operator dependence [69]. Ultromics’ EchoGo Core, and Core Pro for CHD prediction, provides an AI-enhanced strain analysis and automated LVEF assessment, improving reproducibility and holding regulatory approval in various regions. Bay Labs’ EchoMD AutoEF provides AI-assisted automated quantification of LVEF. Us2.ai offers an AI-powered echocardiography analysis platform for automated cardiac phenotyping and guideline-based reporting. Collectively, these platforms have analyzed millions of echocardiograms, supporting feasibility and clinical utility in practice [66,67]. Current FDA-cleared applications include automated view classification, LVEF measurement, myocardial strain quantification, and image quality assessment [66,67]. Emerging precision applications, including HFpEF phenogrouping, cardiotoxicity prediction, and cardiomyopathy risk stratification, hold transformative potential for personalized cardiac care, but their integration into routine practice will depend on rigorous prospective validation and regulatory endorsement [70].

### 4.2. Proposed Practical Implementation Framework

**Figure 3:**
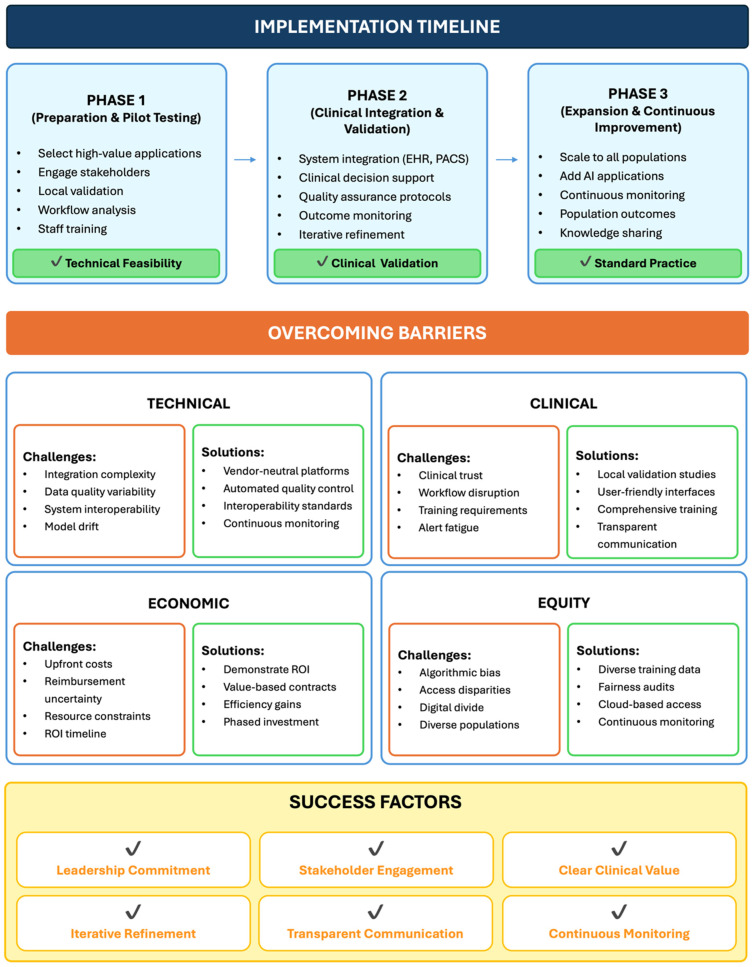
Proposed Practical Implementation Roadmap. Implementation of precision echocardiography requires a phased approach with attention to technical, clinical, economic, and equity barriers. Phase 1 focuses on pilot testing with local validation and workflow establishment. Phase 2 integrates AI into clinical systems with decision support and outcome monitoring. Phase 3 expands to additional applications and scales across populations.

Integrating precision echocardiography into clinical practice requires an evidence-based, phased implementation framework that addresses technical, clinical, and organizational challenges (Figure 3) [66,71,72,73].

Phase 1 (Preparation & Pilot Testing):

Implementation begins with the selection of high-value use cases that address clear clinical needs and yield measurable outcomes (e.g., HFpEF phenogrouping or cardiotoxicity prediction [71]. Early engagement of stakeholders—including cardiologists, cardiac sonographers, and IT staff—supports organizational buy-in and helps identify barriers early [72,73]. Technical validation in local cohorts is essential because AI performance may vary with population characteristics, imaging equipment, and acquisition protocols [74,75]. Workflow analysis should determine the most effective integration points, while staff training supports technical proficiency and shared understanding of AI capabilities and limitations [71,72,73]. Key evaluation metrics include technical performance (e.g., sensitivity, specificity, reproducibility), workflow efficiency, and user acceptance.

Phase 2 (Clinical Integration & Validation):

AI tools are incorporated into routine workflows with systematic monitoring to ensure consistent performance. Integration with echocardiography systems, electronic health records, and PACS enables automated data transfer and interoperability [66,72]. Clinical decision support should present findings in standardized formats, conveying results and confidence levels while minimizing alert fatigue [71]. Quality assurance protocols should include regular audits, monitoring failure rates, and surveillance for model drift due to changes in population characteristics, imaging equipment, or acquisition protocols [72,74,75]. Outcome monitoring should evaluate process metrics (e.g., utilization rate, time to diagnosis) and clinical endpoints (e.g., diagnostic accuracy, changes in management, patient outcomes [72,73]. Ongoing multidisciplinary collaboration supports iterative workflow refinement.

Phase 3 (Expansion & Continuous Improvement):

After clinical value is demonstrated, institutions can expand validated applications to broader populations and incorporate complementary AI functionalities [66,72]. Continuous quality monitoring sustains performance through ongoing validation and early issue identification. Population-level evaluations can assess the institutional impact on diagnostic accuracy, resource utilization, and patient outcomes, supporting value-based adoption [71,72,73]. Knowledge sharing through publications and collaborative networks can facilitate dissemination of lessons learned. Collectively, this phased framework aims to optimize adoption by systematically addressing technical, clinical and organizational challenges.

### 4.3. Overcoming Barriers

Identifying and addressing barriers to implementation is essential for translating precision echocardiography into routine clinical practice (Figure 3) [72].

Technical barriers (e.g., integration complexity, variable data quality, and limited interoperability) may be addressed through vendor-neutral platforms, cloud-based processing, automated quality control, and adherence to interoperability standards.

Clinical barriers (e.g., limited clinician trust, workflow disruption, training demands) may be addressed through local validation studies, user-friendly interfaces, and comprehensive education programs.

Economic barriers (e.g., upfront costs, reimbursement uncertainty, resource constraints) may be addressed by demonstrating return on investment, establishing value-based contracts, and documenting efficiency gains.

Equity barriers (e.g., algorithmic bias, access disparities, the digital divide) may be addressed through diverse training datasets, cloud-based access strategies, and continuous fairness audits [76,77].

Ultimately, successful implementation depends on leadership commitment, early clinician engagement, clear demonstration of clinical value, iterative workflow refinement, transparent communication, and ongoing monitoring to ensure safety, equity, and effectiveness.

## 5. Future Directions

The trajectory of precision echocardiography points toward a future in which cardiovascular assessment becomes increasingly comprehensive, integrated, and widely accessible. Expected developments include expanded FDA approvals for precision applications, seamless integration with electronic health records, real-time clinical decision support at the point of care, and outcome studies demonstrating clinical benefit and real-world impact. Multiple prospective trails (e.g., NCT07010952, NCT06310330) are currently registered on https://clinicaltrials.gov (accessed on 21 February 2026) to evaluate AI-enabled precision echocardiography applications with results anticipated in the coming years. Future directions also include genomic–echocardiography integration for precision cardiomyopathy management [19], multimodal AI combining echocardiography with ECG, biomarkers, and other imaging modalities [19,20], point-of-care precision using portable echocardiography devices with cloud-based AI [78], and population-level precision portable echocardiography-based screening programs to identify high-risk individuals [79,80]. Ultimately, these advances support a vision of routine precision echocardiography for all patients, predictive and preventive cardiology that identifies disease years before symptom onset, continuous monitoring via wearable devices, and fully personalized cardiovascular care based on individual phenotypes, genetics, and risk profiles.

## 6. Conclusions

Precision echocardiography represents a paradigm shift from population-average-based to personalized patient-specific cardiovascular assessment enabled by AI. By moving beyond conventional metrics toward comprehensive phenotyping, AI can support earlier detection of subclinical disease, more accurate risk stratification, and personalized treatment selection. While challenges remain, the trajectory of precision echocardiography is clear. Commercial AI platforms are already entering clinical practice, early adopters are demonstrating meaningful value, and the evidence base continues to expand. The question is no longer whether precision echocardiography will become a standard of care, but how efficiently barriers can be addressed to ensure equitable access and benefit across diverse patient populations.

Realizing the full potential of precision echocardiography necessitates coordinated efforts among multiple stakeholders. Rigorous validation of AI tools across diverse populations and systematic attention to algorithmic fairness are essential. Clinicians must actively engage with these technologies, contribute to their optimization, and promote adoption within clinical practice. Health systems must invest in necessary infrastructure, support integration, and ensure equitable patient access. Industry partners should prioritize clinical utility and interoperability rather than proprietary solutions. Regulatory bodies must balance patient safety with facilitation of innovation through adaptive frameworks.

Ultimately, the success of precision echocardiography will depend on sustained collaboration, innovation, and a shared commitment to equity, ensuring that these advances translate into improved cardiovascular outcomes for all patients.

## Data Availability

No new data were created or analyzed in this study. Data sharing is not applicable to this article.
